# Comparing Salt Tolerance at Seedling and Germination Stages in Local Populations of *Medicago ciliaris* L. to *Medicago intertexta* L. and *Medicago scutellata* L.

**DOI:** 10.3390/plants9040526

**Published:** 2020-04-19

**Authors:** Sonia Mbarki, Milan Skalicky, Pavla Vachova, Shokoofeh Hajihashemi, Latifa Jouini, Marek Zivcak, Pavel Tlustos, Marian Brestic, Vaclav Hejnak, Aziza Zoghlami Khelil

**Affiliations:** 1 Department of Botany and Plant Physiology, Faculty of Agrobiology, Food and Natural resources, Czech University of Life Sciences Prague, Kamycka 129, 16500 Prague, Czechia; vachovap@af.czu.cz (P.V.); marian.brestic@uniag.sk (M.B.); hejnak@af.czu.cz (V.H.); 2 Laboratory of Valorisation of Unconventional Waters, National Institute of Research in Rural Engineering, Water and Forests (INRGREF), BP 10, Ariana 2080, Tunisia; 3 Plant Biology Department, Faculty of Science, Behbahan Khatam Alanbia University of Technology, Khuzestan 47189-63616, Iran; hajihashemi@bkatu.ac.ir; 4 Animal Production and Forage Laboratory, National Institute of Agronomic Research of Tunisia, Hedi Karray Street, El Menzeh 1004, Tunisia; jouini.latifa2016@gmail.com (L.J.); zoghlami.aziza@gmail.com (A.Z.K.); 5 Department of Plant Physiology, Slovak University of Agriculture, Nitra, Tr. A. Hlinku 2, 94901 Nitra, Slovak Republic; marek.zivcak@uniag.sk; 6 Department of Agroenvironmental Chemistry and Plant Nutrition, Faculty of Agrobiology, Food and Natural resources, Czech University of Life Sciences Prague, Kamycka 129, 16500 Prague, Czechia; tlustos@af.czu.cz

**Keywords:** alfalfa, snail medick, salt stress, tolerance index (TI), proline, soluble sugar, sensitivity index (SI)

## Abstract

Salt stress is one of the most serious environmental stressors that affect productivity of salt-sensitive crops. *Medicago ciliaris* is an annual legume whose adaptation to agroclimatic conditions has not been well described. This study focused on the salinity tolerance of *M. ciliaris* genotypes compared to *M. intertexta* and *M. scutellata* in terms of plant growth, physiology, and biochemistry. Salt tolerance was determined at both germination and early seedling growth. Germination and hydroponic assays were used with exposing seeds to 0, 50, 100, 150, and 200 mM NaCl. Among seven genotypes of *M. ciliaris* studied, *Pop1*, *355*, and *667*, were most salt tolerant. Populations like *355* and *667* showed marked tolerance to salinity at both germination and seedling stages (TI ≤1, SI_(_FGP_)_ > 0 increased FGP *≥* 20% and SI_(_DW_)_ < 0 (DW decline ≤ 20%); at 100 mM); while *Pop1* was the most salt tolerant one at seedling stages with (TI =1.79, SI_(_FGP_)_ < 0 decline of FGP ≤ 40% and with increased DW to 79%); at 150 mM NaCl). The genotypes, *306*, *773*, and *M. scutellata*, were moderately tolerant to salt stress depending on salt concentration. Our study may be used as an efficient strategy to reveal genetic variation in response to salt stress. This approach allows selection for desirable traits, enabling more efficient applications in breeding methods to achieve stress-tolerant *M. ciliaris* populations.

## 1. Introduction

In recent decades, the cumulative effects of climate change have led to water shortages, soil pollution and increased soil and water salinization. Loss of available arable land and increased human population are major threats for agricultural sustainability [1]. It is expected that salt pollution will be one of the main limiting factors for increasing crop production in the future through expansion of cultivated areas [2]. High salt levels in soils or irrigation water represent major environmental concerns for agriculture worldwide and are one of the main abiotic stresses limiting crop growth [3,4]. Excess salinity affects more than 6% of the land, while on irrigated lands, it is over 40% [5]. Soils in affected areas contain high concentrations of soluble salts, mainly NaCl, but also Na_2_SO_4_, CaSO_4_, and KCl [6]. Increased Na^+^ and Cl^–^ in the root zone impairs metabolic processes and damages the photosynthetic apparatus, decreasing photosynthetic efficiency and plant growth [7]. Salt accumulation in soils changes plant physiology and metabolism, and negatively affects germination, seedling growth, vegetative phase, flowering, and fruiting leading to decreased yields and quality of production [8].

Recent advances in research provide great opportunities to develop effective strategies to improve crop salt tolerance and yield in saline soils. Investigating the tolerance of crops species and varieties to NaCl may contribute to the development of practical, cost-effective solutions for managing salt-affected soils [9,10,11]. Saline soil may contain excess Na^+^, Cl^−^, and SO_4_^2-^ that affect plant growth through osmotic stress and ionic toxicity causing an imbalance in plant nutrient uptake and metabolism [4]. Pinpointing the genotypes of new salt-tolerant crop varieties that may outperform current varieties is a pressing need for agriculture biotechnology [4,10]. Many research studies have focused on selecting salt-tolerant genotypes and varieties [8,11,12,13], and considerable improvements in crop growth and yield have been reported using these approaches. However, choosing suitable selection criteria while accounting for complex tolerance mechanism and the diverse response plants exhibit at various developmental stages can hinder the identification of useful genotypes. The development of salt-tolerant plants is still at an early stage but should become more efficient as greater knowledge of the mechanism of salt tolerance is acquired [11]. Adaptation to salt has been studied in numerous plant species, varieties, and halophytes [4,10,14], and the mechanisms have proven to be complex [14]. The use of better-suited plant varieties should improve productivity in marginal areas affected by salinity [4,8,11], which is especially important where crops are irrigated with water containing high concentrations of ions [15]. Poor crop yields often result because the salt tolerance of the varieties grown has not been taken into account. Therefore, the selection of genotypes for better salt tolerance in crops have become a critical requirement for the future of agriculture in arid and semiarid regions [9,16]. Increasing knowledge of the adaptive mechanisms and the morphological, physiological, and biochemical strategies for coping with salt stress are critical for success in crop improvement [10,17]. In the Mediterranean basin, growing legumes is of prime importance because of their agronomic, economic, and nutritional benefits [12,17], and several studies have shown that perennial legumes such as alfalfa (*Medicago sativa*) can be grown successfully in saline marginal areas [7]. Annual *Medicago* are widely distributed throughout the world and can be planted in pastures and dry lands [12]. Since the Mediterranean area includes arid and semi-arid regions, the climatic conditions of these countries are very critical in crop production. Annual *Medicago* can be grown as range plants for animal feeding in dry areas and rain-fed farming systems [12,18].

Salt tolerance is especially important during germination where high soil salinity near the soil surface can inhibit growth [19,20]. Germination is a critical step in a plant’s development, influencing the early growth of the seedling and its relation to the environment and productivity. Salinity stress will reduce plant growth, but plants differ widely in their sensitivity to salts depending on the concentration and duration of exposure [8,20]. Sodium chloride in soil or irrigation water affects germination of glycophytes in two ways: by decreasing germination rate and capacity of germination [21,22]. The reduction in germination is due to the increase in the osmotic pressure of the soil solution, which delays imbibition and limits the water absorption required for metabolism [22]. During germination, the salinity effects can be manifested firstly as osmotic (reversible) and secondly as toxic (irreversible) [23]. Thus, the seed will be rehydrated as soon as it is placed in soil containing sufficient moisture.

Medicago are well adapted to many soil types ranging in texture from sandy loam to clays. The increment of annual Medicago as *Medicago ciliaris* into farming system in dry land is a possible mean to slow or stop the spread of dryland salinity. The selection of tolerant populations to salt stress and selecting near isogenic lines are a valuable resource for genetic studies which can be introduced in green farming systems.

To select salt-tolerant varieties, it is necessary to establish an effective screening method for identification of useful salt tolerance parameters for analysis at the molecular level [14,16,24]. An understanding of the responses of different crops to adverse conditions at different growth stages is essential for selecting spices or varieties able to sustain crop production under abiotic stress [16,25]. The goal of this investigation was to determine the effects of salinity stress on spontaneous forage legumes at germination and early growth stages through physiological characterization. We compared the seed germination and the early seedling growth of seven populations of *Medicago ciliaris* to one population of *M. intertexta* and one population of *M. scutellata*. The aim was to select salt-tolerant ecotypes of annual *Medicago* species that can be sown on saline soil or after using salt irrigation in order to restore soil fertility and reclaim marginal lands.

## 2. Results

### 2.1. Effect of Salt on Seed Germination

The effects of salt stress on seed germination of seven populations of *Medicago ciliaris* and those of *Medicago intertexta* and *Medicago scutellata* were studied, and the main results are discussed below.

#### 2.1.1. Salt Stress Effect on Final Germination Percentage (FGP)

In controls without salt treatment, the FGP varied from 62.5% in 667 to 100% in *Pop2*, *Pop3*, and *M. intertexta (M. int)*, showing a significant variability among the populations. In the presence of NaCl, the FGP decreased significantly depending on the concentration: 56.7% at 50 mM NaCl in *773* and 21% at 200 mM NaCl in *355*, *M. scut*, and *Pop1*. At 150 mM NaCl, the FGP was > 50% in *Pop2*, *Pop3*, and *355* of *M. ciliaris* and *M. scut*, indicating a better capacity for germination under saline conditions compared to the other populations (Table 1). Analysis of the two-factor classification variance (salt treatment, populations) showed highly significant effects of salt treatment, populations, and their interaction on the final germination percentage of all populations (Table 2).

#### 2.1.2. Salt Stress Effect on Germination Rate (T50)

Analysis of variance with two factors (salt treatment, populations) showed a significant effect (*p* < 0.05) of salt and populations on the germination rate (T50), but their interaction was not significant (*p* > 0.05) (Table 3). The T50 varied among populations and increased with increasing salt concentration while the level varied from 1.9 days at 50 mM to 2.3 days at 200 mM of NaCl (Table 3). In absence of salt, the T50 varied from 1.2 days in *Pop2* and *773* to 2 days in *667*. At 100 mM, the highest value of T50 was observed in *Pop3* and *306* populations; hence, at 150 mM and 200 mM of NaCl, the T50 increased for the majority of populations (except *Pop1* and *355* at 200 mM). The average T50 was significantly higher at 150 mM (2.6 days) compared to that of the control (1.7 days). Hence, *Pop3*, *355*, and *M.* *scut* reached 50% of germination (T50) after >3 days at 150 mM.

Salt changes the osmolarity of the soil which affects the ability of the seeds to imbibe water, thus delaying germination.

#### 2.1.3. Sensitivity Index to Salt Stress for Seed Germination (SI)

**Figure 1:**
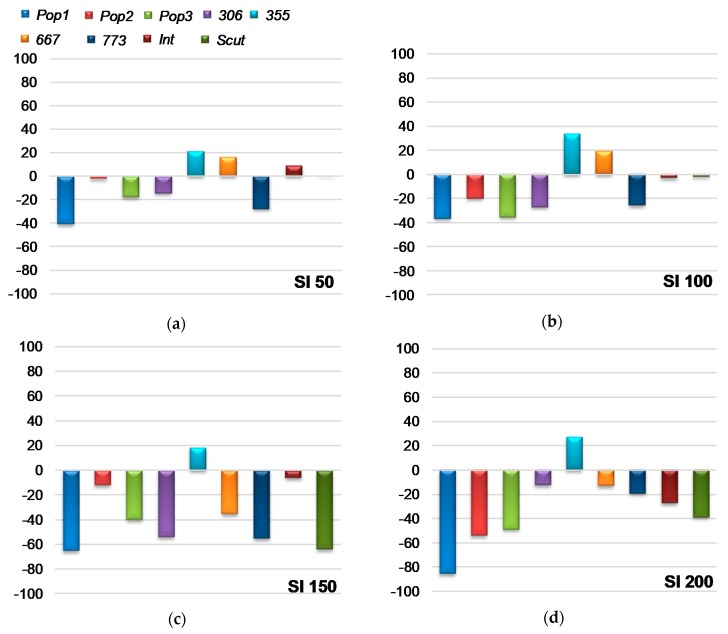
Effect of salt stress (0, 50, 100, 150, 200 mM NaCl) on salt sensitivity index (SI) for final germination percentage (FGP), noted SI_(_FGP_)_, of *M. ciliaris* populations (*Pop1, Pop2, Pop3, 306, 355, 667, 773*) compared to one population of *M. intertexta* (*M. int*), and one population of *M. scutellata* (*M. scut*). The values are the average of 10 seeds replicated 5 times.

For a better screening of populations to salt stress tolerance at germination stage, we calculated the sensitivity index using the germination rate at all salt concentrations. Figure 1 showed a negative SI_(FGP)_ for all populations at all salt concentration except for *355* which had a positive SI (≥ 20%) at all treatments and *667* at only 50 and 100 mM. The percentage of reduction of germination rate did not exceeded 35% for these populations (Figure 1).

### 2.2. Hydroponic Culture Assay

#### 2.2.1. Effect of NaCl on Growth

Responses of plants to salt stress is a result of the complex interaction among different morphological, physiological, and biochemical processes [26]. Globally, there is a genotypic difference in response of legume species and cultivars to salt stress. Our study showed a wide variation between populations in terms of dry weight, epicotyl, and radicle length in control and under salt stress conditions. Under salt stress, dry weight was lower than controls in all populations. Overall, the two-factor (treatment, population) ANOVA showed a highly significant effect of salt treatment and population on growth in terms of plant biomass, epicotyl, and radicle length (Table 4).

**Figure 2:**
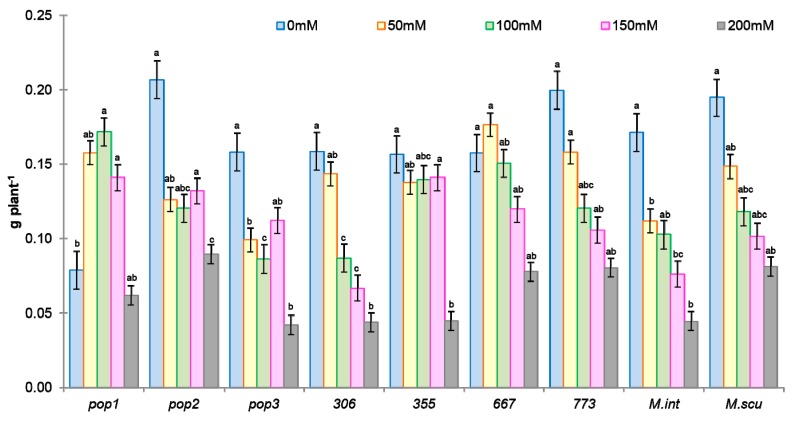
Effect of different concentrations of salt stress (0, 50, 100, 150, 200 mM NaCl) on DW (g plant^−1^) of the seven populations of *M. ciliaris* (*Pop1, Pop2, Pop3, 306, 355, 667, 773*) compared to one population of *M. intertexta* (*M. int*) and one population of *M. scutellata* (*M. scut*) The means are averages of 25 plants (5 plants/*n* = 5 repetitions for each salt treatment). The standard deviation annotated by different lower-case letters in the same stress type indicate significant differences at *p* < 0.05, Tukey’s post hoc test.

The ranking of populations according to their tolerance to NaCl showed a reduction of dry weight with increasing salt concentrations (Figure 2).

In control without salt, the plant dry weight was variable between populations, it was high in *Pop2*, *773*, and *M. scutellata* (Figure 2). In the presence of salt, the plant dry weight was very variable; it increased in some populations (*Pop1*, *667*) and decreased in others (*Pop2*, *Pop3*, *306*, *773*, *M. int*, and *M. scut*). Salt stress significantly affected the biomass in *M. int* and *M. scut*. With the application of low doses of salt, a decrease in the weights of plants was observed in all populations except in *Pop1*, *355*, *667*. The variability in biomass production is essential for the selection of salinity-tolerant populations that can enhance recovery of saline soil fertility.

The classification of the different populations according to their tolerance to NaCl showed a regression of the biomass, length of epicotyl, and length of radicle (Figure 2). However, the largest reductions were noted at 50 mM NaCl in *Pop2* (50%) and in all populations at higher NaCl concentrations. On the other hand, *355* and *667* seemed to be less affected by salt, at least for this trait and displayed the lowest values of reduction. *Pop1* did not show a reduction for this parameter, but there was an improvement. The percentage of reduction in this case for all populations varied from 10% at 50 mM to 50% at 100 mM for all populations, and to 50% or more for *M. scut* and *M. int* at 150 mM NaCl and higher. The same reduction was also found for all populations of *M. ciliaris* at 200 mM NaCl (Figure 2).

**Figure 3:**
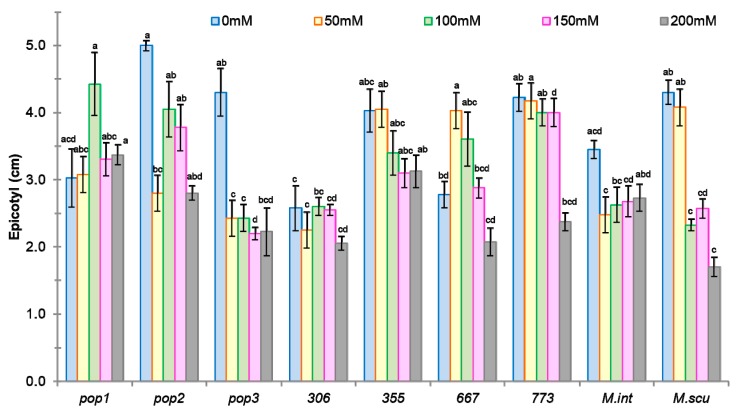
Effect of different concentrations of salt stress (0, 50, 100, 150, 200 mM NaCl) on epicotyl length (cm) of seven populations of *M. ciliaris* (*Pop1, Pop2, Pop3, 306, 355, 667, 773*), compared to one population of *M. intertexta* (*M. int*) and one population of *M. scutellata* (*M. scut*). The means are averages of 25 plants (5 plants/*n* = 5 repetitions for each salt treatment). The standard deviation annotated by different lower-case letters in the same stress type indicate significant differences at *p* < 0.05, Tukey’s post hoc test.

**Figure 4:**
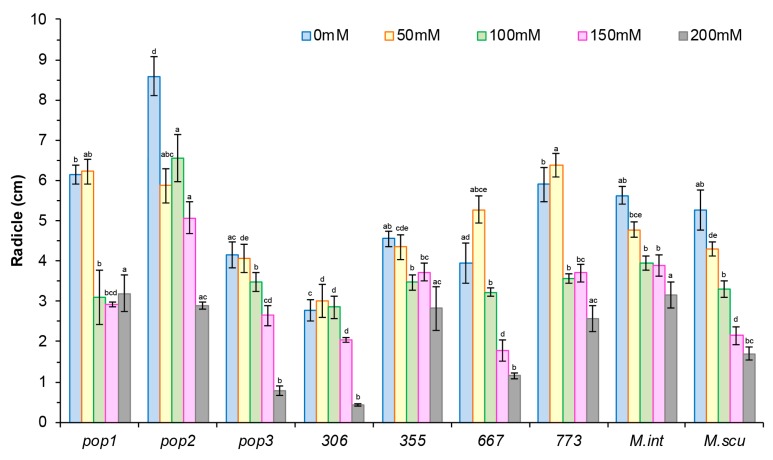
Effect of different concentrations of salt stress (0, 50, 100, 150, 200 mM NaCl) on radicle length (cm) of seven populations of *M. ciliaris* (*Pop1, Pop2, Pop3, 306, 355, 667, 773*), compared to one population of *M. intertexta* (*M. int*) and one population of *M. scutellata* (*M. scut*). The means are averages of 25 plants (5 plants/*n* = 5 repetitions for each salt treatment). The standard deviation annotated by different lower-case letters in the same stress type indicate significant differences at *p* < 0.05, Tukey’s post hoc test.

As roots are in direct contact with the soil solution, the root and shoot length provides an important clue to the response of plants to salt stress. A reduction in epicotyl and radicle length at all salt doses was noted in all populations except in *Pop1*, *355*, and *667*, where the reduction did not occur until salt concentrations reached 100 mM NaCl (Figure 3 and Figure 4).

The ranking of the different populations according to their tolerance to salinity showed a regression of the epicotyl length of the plantlet for the different populations studied (Figure 3). However, *Pop1*, *667*, and *355* were less affected by salt, at least for this feature, and displayed the lowest values for plant growth reduction, between 10% and 30%, at all NaCl doses. In the case of population *Pop1*, this parameter seemed to be not affected by salt concentration.

Increasing salt concentration in Hoagland’s solution decreased significantly the seedling root length (RL) in all populations (Figure 4). For radical length, a large variation between populations was observed in absence and in presence of salt (Figure 4). In control conditions, *Pop2* had the longest radical while in presence of salt, *773* had the longest radical at 50 mM NaCl. The highest reduction of radical length was observed in *Pop1* and *773* (50%) at the highest concentrations (100 and 150 mM) while the reduction is less in *Pop3, 306*, and *355*.

#### 2.2.2. The Sensitivity Index to Salt Stress for Growth (SI), Noted SI_(DW)_

**Figure 5:**
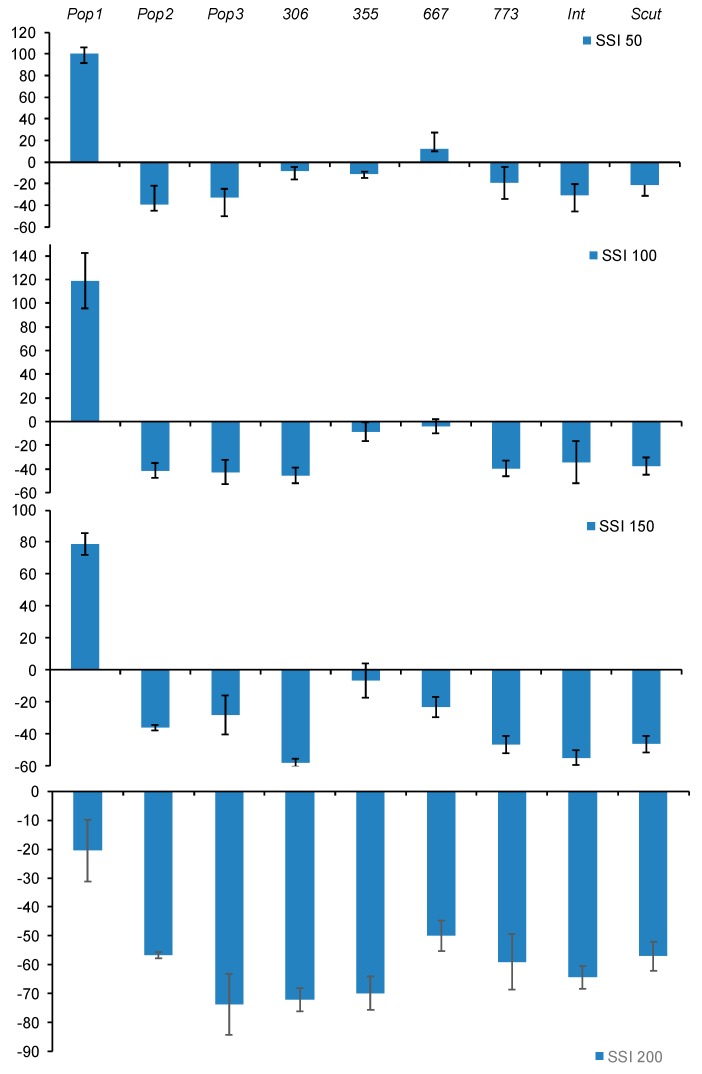
Effect of salt stress (0, 50, 100, 150, 200 mM NaCl) on sensitivity index SI (*SI_(__DW)_*) for growth of seven populations of *M. ciliaris* (*Pop1, Pop2, Pop3, 306, 355, 667, 773*), compared to one population of *M. intertexta* (*M. int*), and one population of *M. scutellata* (*M. Scut*). The means are averages of 25 plants (5 plants/*n* = 5 repetitions for each salt treatment). The error bars show standard deviation; *p* < 0.05.

To select the populations for their tolerance to salt stress, we calculated the index of sensitivity (under hydroponic conditions) using the dry weight of the plants after growth at 50, 100, 150, and 200 mM NaCl (Figure 5). At all NaCl concentrations, *Pop1* had a positive sensitivity index SI_(DW)_ (SI ≥ 80% for salt until 150 mM NaCl), showing good tolerance to salinity at the young seedling stage compared to other populations. Populations of *355* and *667* appeared to be moderately tolerant, with a positive sensitivity index up to 100 mM NaCl. Populations of *M. int* and *M. scut* were less tolerant than populations of *M. ciliaris* at 100 mM NaCl or greater.

The calculations of plant biomass production throughout the experimental period revealed that *Medicago ciliaris* populations, as well as *M. int* and *M. scut* were able to maintain growth at all salinity levels and even increased it for some populations like *Pop1* at low NaCl concentrations (Figure 2). The values of SI_(DW)_ for *Pop1* were positive (Figure 5), indicating an enhanced growth compared to control plants and showing that *Pop1* is the most tolerant population of *Medicago ciliaris* compared to others at the seedling stage, and this is probably due to its origin from saline soil.

#### 2.2.3. The Salt Tolerance Index at Early Growing Stage (TI)

**Figure 6:**
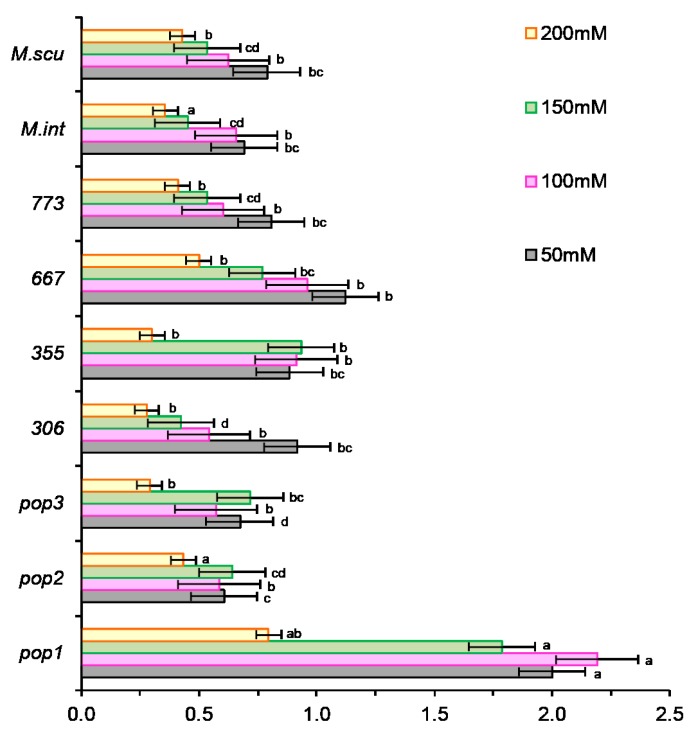
Effect of salt stress (0, 50, 100, 150, 200 mM NaCl) on tolerance index (TI). Tolerance index of Pop1, *Pop2, Pop3, 306, 355, 667,* and *773*, compared to one population of *M. intertexta* (*M. int*) and one population of *M. scutellata* (*M. scut*). The means are averages of 25 plants (5 plants/*n* = 5 repetitions for each salt treatment). The standard deviation annotated by different lower-case letters in the same stress type indicate significant differences at *p* < 0.05, Tukey’s post hoc test.

The one-way ANOVA revealed significant differences among the studied populations of *M. ciliaris* in all growth parameters measured with respect to their salt tolerance index and for different NaCl concentrations (*p* > 0.05). *Pop1* had the highest tolerance index (TI) at all salinity levels, almost two times that of the rest of populations, while *667* and *355* showed intermediate values (Figure 6).

### 2.3. Biochemical Parameters

#### 2.3.1. Chlorophyll a Content

Analysis of two-way ANOVA (treatment, population) showed a significant effect of salt stress and population (*p* < 0.0001 and *p* < 0.0001 respectively) as well as their interaction on Chl *a* content (Table 5). In the absence of salt, the Chl *a* content varied from 0.3 mg g^−1^ FW in *355* and *M. int* to 2.3 mg g^−1^ FW for *306*. The population *306* was the richest population in Chl *a*. In general, the value decreased at 50 mM NaCl compared to the control, but there was some increase at 100 mM in most populations. The lowest values were observed at 150 and 200 mM NaCl in all population except that of *M. scut* which had lower Chl *a* content in all salt treatments, most likely due to its naturally occurring yellow leaf colorant; this is related to the yellow leaf color of population M. scut in nature.

#### 2.3.2. Chlorophyll b and Total Chlorophyll Content

The population *667* was the richest in Chl *b*. Salt stress (50 mM) decreased Chl *b* in all populations except *Pop2* and *306* and increased it at 200 mM in all populations except in *Pop3* (Table 5). Two-way ANOVA analysis of Chl *b* and total chlorophyll content demonstrated that the salt stress effect was highly significant. The effect of population and interaction with salt concentrations was also highly significant (*p* < 0.01). Populations *Pop3* of *M. ciliaris* and *M. scut* presented the lowest values of Chl *a*, Chl *b*, and total chlorophyll (Table 5).

#### 2.3.3. Proline Content

**Figure 7:**
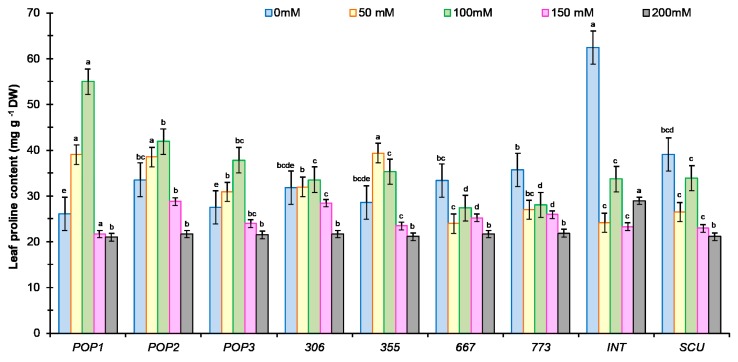
Effect of salt stress concentrations (0, 50,100, 150, 200 mM NaCl) on proline content in leaves for seven populations of *M. ciliaris* (*Pop1, Pop2, Pop3, 306, 355, 667, 773*), one population of *M. intertexta* (*M. int*), and one population of *M. scutellata* (*M. scut*). The means are averages of *n* = 5. The standard deviation annotated by different lower-case letters in the same stress type indicate significant differences at *p* < 0.05 according to Tukey’s post hoc test.

Proline content was measured in leaf tissue at an early vegetative stage to determine its association with salt tolerance. Two-way ANOVA analysis (salt stress, population) showed a significant effect of salt and population on proline content (*p* < 0.0001) and their interaction (*p* < 0.001). The proline content increased in some populations (*Pop1*, *Pop2*, *Pop3*, and *355*) under saline conditions (Figure 7), while in *667*, *773, M. scut*, and *M. int*, salt stress decreased proline content independently of NaCl concentration. There was no significant change in proline content for *306* under low salt stress dose (Figure 7). In controls, *M. int* had the highest proline content. Up to 150 mM, proline levels were comparable to those of the control (Figure 7). Above 150 mM, proline content in leaves decreased in all populations, with *Pop2* and *306* showing the highest values. The lowest values were observed at 200 mM NaCl in all populations. Positive correlation was found between proline accumulation and plant weight (r = 0.43; *p* = 0.006; *n* = 38). The more vigorous growers accumulated more proline. Soil salinity causes osmotic stress, which induces the synthesis of several amino acids, including proline in glycophytes.

#### 2.3.4. Sugar Content

**Figure 8:**
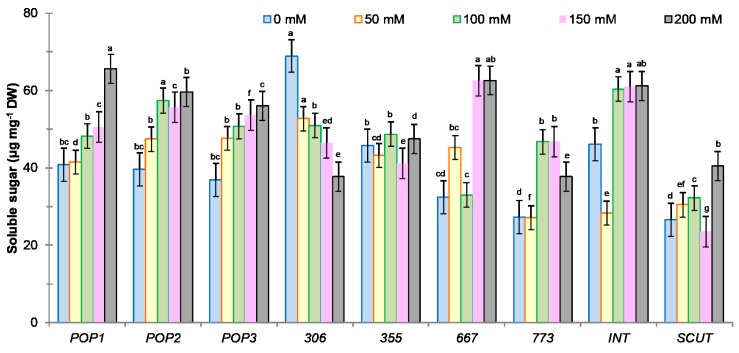
Effect of salt stress concentrations (0, 50, 100, 150, 200 mM NaCl) on leaf sugar content for seven populations of *M. ciliaris* (*Pop1, Pop2, Pop3, 306, 355, 667, 773*), compared to one population of *M. intertexta* (*M. int*) and one population of *M. scutellata* (*M. scut*). The means are averages of *n* = 5. The standard deviation annotated by different lower-case letters in the same stress type indicate significant differences at *p* < 0.05 according to Tukey’s post hoc test.

ANOVA two-way (salt stress, population) analysis showed a significant effect of salinity and population on sugar content (*p* < 0.0001) and their interaction effect (*p* < 0.001). Carbohydrate content in salt-stressed plants increased in all populations of *M. ciliaris* (except in *306* and *355)* and *M. int* and was dependent of salt concentration. While for *M. scut*, sugar content increased significantly at 200 mM (Figure 8), in population *306*, sugar content decreased with increasing salinity, and in *355*, there is no significant change in sugar content according to salinity (Figure 8). A positive correlation between sugar content and plant dry weight (DW) (r = 0.37; *p* = 0.03; *n* = 32) was observed.

#### 2.3.5. PCA Analysis for Traits

**Figure 9:**
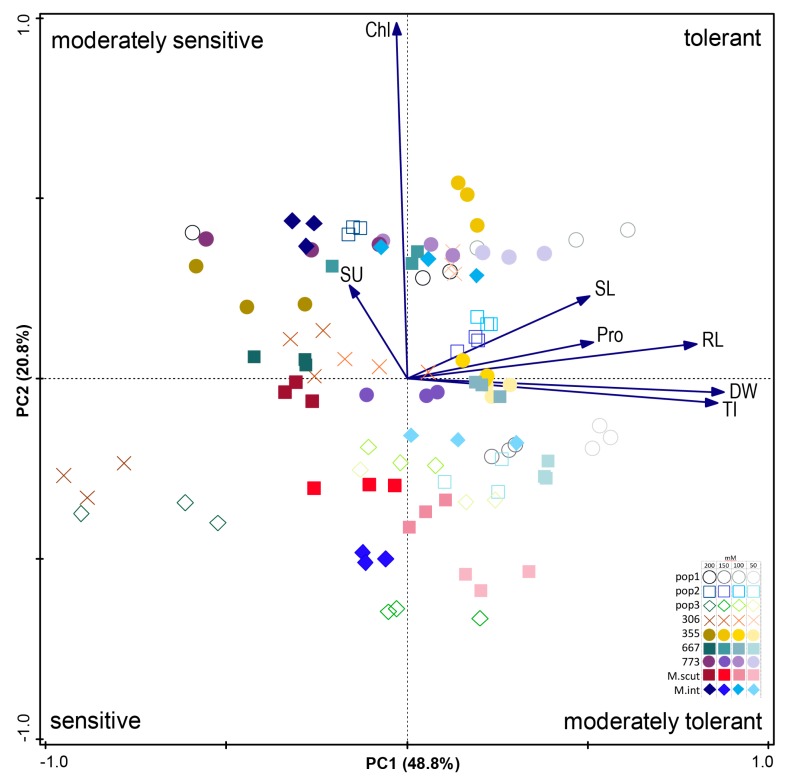
Ordination diagram (Principal Component Analysis). The bi-plot displays the populations and measured properties of plants. The first and second axes together explain 69.6% of variance. The concentrations of salt stress (0, 50, 100, 150, 200 mM NaCl) are shown by the color intensity of the symbol that represents the type of population (table in graph). Chl—chlorophyll; SU—sugar; SL—epicotyl; Pro—proline; RL—radicle; DW—dry weight; TI—Tolerance Index.

The PCA analysis revealed variation among the traits for each factor, and a loading value above 0.5 was considered significant. The first component (PC1) and second component (PC2) explained 69.6% of the total variance, and data were used to make a score plot (Figure 9). NaCl concentrations less than 150 mM are on the right side of the score plot, and the populations are closely spaced, indicating that their responses to salinity were similar. This is attributable to their similar geographic origin (north Tunisia), climate, and environmental conditions and confirms that the environment of origin influences the effect of salinity on adaptation among different genotypes. The loading plot of all the measured variables included in the PCA is shown in Figure 9. The variables with the highest scores in PC1 at positive side, are plant biomass production (DW), tolerance index (TI), epicotyl length (SL), radicle length (RL), and proline content (Pro). In PC2, the highest score was for chlorophyll content (Chl) and sugar content (SU). The populations on the right side at top and bottom were tolerant to moderately tolerant: *Pop1, 355*, and *667* at 50, 100, 150 mM NaCl. Pop *306*, *773*, and *M. scut* were the least tolerant populations compared to other populations of *M. ciliaris* and dependent on salt doses.

## 3. Discussion

In this research, we studied the effect of salt stress on physiological and biochemical parameters in some populations of *Medicago ciliaris* compared to populations of *M. intertexta* and *M. scutellata*. Analysis of the final germination percentage (FGP) and germination rate (T50), showed significant variability among populations with respect to salt concentration. These results are consistent with those of [27] who showed significant variability in salinity tolerance in *Phaseolus* at the germination stage. A study of the salt tolerance of ten native and six exotic potato genotypes in Bangladesh measured stress tolerance trait indices (STTIs) in four groups—tolerant, moderately tolerant, sensitive, and very sensitive—and the data were useful for improving potato yield [28]. FGP decreased significantly depending on salt concentration: 56.7% at 50 mM NaCl in 773 and 21% at 200 mM NaCl in *355*, *M. scut* and *Pop1*. Salt stress from 150 mM NaCl affected FGP in most populations except *Pop2*, *355*, and *M. int* which can maintain an FGP > 50%, thus showing a good capacity for germination in the presence of salt. However, above 100 mM, this rate dropped to less than 67.5% for some varieties while for others, the germination rate exceeded 70%. The presence of NaCl also resulted in a significant decrease in germination in five legume species [29]. On the contrary, seeds exposed to a low concentration of 25 mM NaCl showed an increase in percent germination and germination index [30]. The coefficients of genotypic determination of six natural populations of a Fabaceae plant (*Stylosanthes humilis*) from three ecogeographic regions were high for five of the populations (except population Tamandaré) both for germination percentage (0.89) and germination rate (0.79), indicating the possibility of selection for salt tolerance in these populations [31]. The germination rate (T50) of our study populations increased with increasing salt concentration and varied among the populations, ranging on average from 1.9 to 2.6 days. It was low at (50-100 mM NaCl) in *Pop1*, *Pop3*, and *306* and did not differ significantly between populations at 150 and 200 mM. However, in two sensitive glycophytes, *Phaseolus vulgaris* and *Glycine max*, salt had a depressive effect that slowed the T50 from 50 mM NaCl with critical thresholds at 150 and 200 mM NaCl [29]. A study of tomato cultivars showed that the genotypes BARI Tomato-2, Mintoo, and Unnoyon were more tolerant to high salt stress in terms of T50 and root and shoot characteristics than other genotypes [32].

Excessive NaCl concentrations may cause seeds to lose viability during salt exposure, thereby slowing the germination process [33,34].

According to [29], the germination rate can be used as an early criterion for selecting salt-tolerant species and populations of legumes. Among our populations, *Pop2*, *355*, *667*, and *M. int* were the most tolerant at germination, with the highest germination percentage and germination ability. However, the kinetics of germination at a concentration of 100 mM NaCl showed a high variability among the genotypes of different populations (data not shown). This genotypic variability, which relates to the speed of germination, is a function of the response to salt stress. Data obtained after ten days of salt exposure indicated that the final seed germination percentage decreased significantly with increasing salinity (Table 1). Thus, not all seeds had the same capacity to tolerate salinity. The genotypes *Pop2*, *355*, *667*, and *M. int* showed positive or near zero sensitivity indices for germination percentage regardless of treatment compared to other populations. The germination tolerance indices (TI) for the three experimental replicates showed that the *355* and *667* genotypes were the most vigorous, while the SI of the others did not exceed 50% until 100 mM NaCl. Applying two-way ANOVA showed a significant effect of genotype and treatment on SI and a highly significant effect of treatment on the final percentage of germination and seedling germination rate (T50). Some populations, such as *Pop1*, were salt stimulated with increased germination speed and with positive SI after pre-treatment salinity stress, retarding germination and increasing the percentage of recovery of seed germination [35].

Growth parameters including plant biomass (DW), epicotyl length, and root length were studied under salt stress condition. The DW of plants from *M. ciliaris*, *M. int*, and *M. scut* populations varied with salinity concentration. A decrease in the biomass of the plants with exposure to salt was noted in most populations (except *Pop1*, *355*, and *667)* whose percentage of reduction in dry weight increased from 50 mM to 200 mM NaCl. At 200 mM, a depressive effect of salt on germination was noted for all population of *Medicago ciliaris*, *M. scut*, and *M. int*. Moreover, a reduction in the length of the epicotyl and the radicle was noted in all populations for all doses of salt, except for *Pop1*, and a slight reduction for *667* and *355*. The detrimental effect of salt occurs at the level of the whole plant and is caused by limiting leaf area expansion to stomatal conductance [36]. In terms of growth, the effect of salt stress is a reduction in biomass and in nutrient homeostasis [37]. This effect is common in glycophytes [38], where the observed decrease in vegetative growth can be explained by an increase in osmotic pressure caused by NaCl, which reduces water absorption by the roots. Plants thus adapt to salt stress by reducing their growth in order to avoid damage [10]. The effects of salinity on the growth of seedlings grown under controlled conditions depend on several factors. They vary according to the NaCl concentration, the species, the provenance, the vegetative stage, and the part of the plant [8]. The effects of salinity are mainly manifested by a slower vegetative growth [37]. The reduction in shoot and root growth by salt stress can be a consequence of unbalanced nutrient uptake by the seedlings; and the inhibition of shoot and root elongation is due to diminished water and essential mineral uptake by plants [15,30]. In general, salt stress inhibits the growth of shoots more than roots. Mahdavi and Sanavy [39] observed significant differences among grass pea (*Lathyrus sativus*) cultivars under salt stress in terms of coleoptile and root growth, indicating that genetic variation exists within the cultivars and that salt inhibited coleoptile growth more than root growth.

Salt sensitivity index (SI) is a reliable parameter for estimation of salt stress level and identification of potential salt-tolerant cultivars. Considering the SI measured in this study, the best results were observed in *355* and *667*, whereas *Pop1* had the highest weight for all salt levels, thus showing good tolerance to salt stress at the young seedling stage compared with other populations. Similarly, the genotypes *355* and *667* had lower yield reduction under stress condition and root growth was maintained under salt stress, which helps populations or genotypes to tolerate salt stress by maintaining shoot growth toward salt dilution, by salt exclusion uptake, limiting Na accumulation in shoots and more vigorous shoot growth [37]. In contrast, [40] showed salt stress affected germination, radicle elongation, chlorophyll content, and Net assimilation, indicating that alfalfa (*Medicago sativa*, China seeds) was relatively sensitive to salt stress during germination and early seedling growth stage.

*Medicago ciliaris* had higher root indices, highlighting the great importance of roots in salt tolerance. The percentage of reduction in dry matter, generally considered as an index of the sensitivity of plants to salt stress, showed that the 100 mM NaCl concentration was insufficient to cause a relative reduction under 50% compared with controls [41]. Even at lower concentrations like 50 mM, many species showed marked reductions.

Screening for salinity tolerance in diverse alfalfa germplasm under non-stress conditions and salt stress (0 mM and 150 NaCl) [42] showed that highly significant variation was found among alfalfa genotypes and salt treatments for fresh and dry matters. Three tolerant genotypes from coastal origin, which displayed the best tolerance, used an Na^+^ “inclusion mechanism” to prevent salt stress. The decrease in growth of aerial parts could be explained by disturbances in tissue growth regulator levels, particularly secondary metabolites, but also by a decrease in the photosynthetic capacity caused by the decrease in stomatal conductance of CO_2_ under salt stress [13]. In all cases, this reduction in the growth of the various aerial parts is considered an adaptive strategy necessary for the survival of plants exposed to salinity [25]. This allows the plant to store the energy needed to cope with stress and to reduce the irreversible damage that occurs when the threshold of lethal concentration is reached [43]. Bukhari et al. [44] showed that, in general, environmental stress affected germination and growth processes of plants.

Analysis of biochemical parameters showed that chlorophyll content (Chl *a*, Chl *b*) varied with population and salt levels. According to [45], a reduction of chlorophyll *a* and chlorophyll *b* was reported in stressed chili plants compared to the control. The reduction of chlorophyll *a* and chlorophyll *b* may be related to the sensitivity of their biosynthesis to sodium chloride, although Chl *b* is less affected. Salt stress plays a role in decreasing chlorophyll activity and inhibits the synthesis of 5-amino-levulinic acid [46]. The reduction of CO_2_ in the leaves is associated with an inhibition of photosynthesis [47].

Our results showed that increasing salt stress increased accumulation of sugars in the leaves, but the effect depended on the population. *Pop1* of *M. ciliaris* accumulated more soluble sugars in the leaves than *M. scut* and *M. int* at 200 mM NaCl. The same result was found for *Medicago sativa*, in which sugar accumulation increased with increasing salt concentration. The concentration of soluble sugars in the leaf tissues of stressed plants is always greater than in stems and plays a dual role in stressed plants as a tolerance characteristic, since they participate in metabolic events and dehydration [48]. According to [49], high levels of osmolyte accumulation in plants were well correlated with stress tolerance through the escape of radicals and protective enzymes. Soluble sugars are indicators of the degree of stress [50]. Because of their significant increase in severe stress, metabolic sugars such as glucose, galactose, sucrose, and fructose are determining factors in the osmotic adjustment for resistance to various stresses [49].

There was an increase in proline in leaves with increasing salt level in populations, except for *M. int*, *M. scut*, *667*, and *773*, which had the lowest proline concentration under salt stress. The highest content was recorded at the 100 mM dose in *Pop1* (saline site geographic provenance, Raoued el Hessian) and to a lesser extent for the content in leaves of *Pop2*, *Pop3*, and *355*. At 200 mM NaCl, the proline content in all populations was comparable. In both control and stressed plants, this amino acid was preferentially concentrated in leaves at significant levels depending on populations. Our results are consistent with those of [39], who showed that proline levels rose with increased salinity level, which correlated to reduction of hypocotyl, radicle length, and dry and fresh weight of different cultivars of grass pea (*Lathyrus sativus*). The accumulated proline could play an osmotic role in the regulation of cytoplasmic pH or constitute a reserve of nitrogen used by the plant after the period of stress [51]. It neutralizes the ionic and osmotic effects of salt accumulation in the vacuole. Increased proline synthesis is considered a stress marker and an adaptive action taken by plants. It acts as a protective soluble compound without exerting a toxic effect as is the case with ions. Proline is involved in the detoxification of active forms of oxygen and the stabilization of proteins [52,53]. Its content is correlated with salt tolerance [54]. The ability to maintain a high concentration of proline in tissues as well as adequate levels of chlorophyll and water content has been suggested for use as tolerance markers for an alfalfa population tested in different geographic sites of Morocco [33].

Under the salt stress conditions of our experiments, it was established that tolerance at the germination stage did not in all cases reflect what happened later at the vegetative and early plant establishment stages. The population *Pop1* was not the most tolerant to salt during germination but showed a significant tolerance towards NaCl at the seedling stage. In contrast, *Pop2* is tolerant at germination stage but is not tolerant at early seedling stage. Pop *355* and *667* were recognized by a very marked tolerance to salinity at the germination stage and retained this performance at the advanced stages. However, germination under salt stress is not sufficient to identify salt-tolerant species [40]. In this context, many authors have shown that the response to salinity varied according to the stage of development of the plant [36]. However, germination and the early stages of growth are the most sensitive phases [40].

The traits examined in this study have shown significant variability for most growth parameters and can be used as selection indices to identify germplasm for salt tolerance. By combining of features across all the studied population, natural diversity allowed them to maintain a good relative biomass and have different mechanisms to resist high salinity. PCA analysis performed on annual *Medicago* populations disclosed that tolerant populations had long epicotyls and radicles and produced more proline, while sensitive ones were rich in sugars. Almost all populations on the right side of the graph were tolerant to 50 to 150 mM NaCl and sensitive to 200 mM, while the populations on the left side were sensitive to 200 mM. This result is accommodated with the PCA score plot (Figure 9), which indicated that *306*, *773*, and *M. scut* were moderately tolerant to sensitive to salt stress, depending on salt stress. Because of their high content of proline and salt tolerance index, *Pop1*, *355*, and *667* can be recommended as the most promising local populations, the seeds of which can germinate under salt stress up to 150 mM NaCl. As the need to adapt to climate change continues, the salt tolerant populations identified in this study should serve as viable forage options and future breeding material for areas with excessive salt in the irrigation water and to reclaim arable crop land that has been lost due to high salt content.

## 4. Materials and Methods 

### 4.1. Plant Genotypes and Seed Sources

The study was carried out at an experimental station in National Institute of Agriculture Research (INRAT), Laboratory of Plant and Animal Production (PAF), Tunisia (36, 87° N | 9, 96° E).

The plants material selected for our study came from seven spontaneous local populations of *Medicago ciliaris* (*Pop1*, *Pop2*, *Pop3*, *306*, *355*, *667*, and *773*), compared to one local population of *M. intertexta* (*M. int*) and one variety of *M. scutellata* (*M. scut*). These populations obtained from the National Institute of Agriculture Research (INRAT), Laboratory of Plant and Animal Production (PAF) were collected in different regions of Northern Tunisia, and a duplicate is being conserved at the National Genes Bank (NGB). Their origins and characteristics have been summarized (Table 6) [55].

### 4.2. Germination Assays

**Figure 10:**
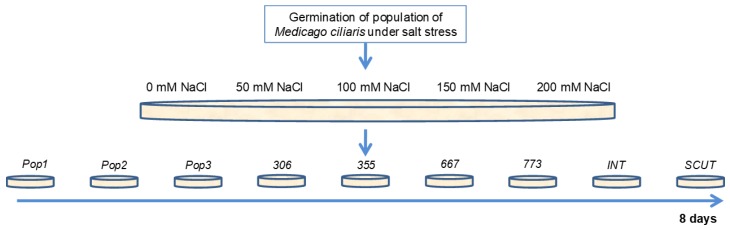
Protocol for germination test of seven populations of *M. ciliaris* (*Pop1, Pop2, Pop3, 306, 355, 667, 773*), compared to one population of *M. intertexta* (*M. int*) and one population of *M. scutellata* (*M. scut*) under salt stress (0, 50, 100, 150, 200 mM NaCl equivalent to 5.9, 11, 16.5, 20 mMohs/cm at Temperature 25 °C).

The experimental design involved nine populations of *M. ciliaris*, *M. intertexta*, and *M. scutellata* (10 seeds from each population replicated 5 times at each treatment of salt stress). Seeds of homogeneous size were scarified with liquid nitrogen in order to facilitate water imbibition. Then they were transferred to Petri dishes (10 seeds/dish) on imbibed-filter paper type Whatman No. 2 moistened with 10 mL distilled water for the control and 10 mL of the saline solution with 50, 100, 150, and 200 mM NaCl (3, 6, 9, 12 g NaCl/L) (Figure 10). To avoid evaporation, the edges of the Petri dishes were tightly sealed with Parafilm and placed in a germination chamber in the dark for 72 h at 22 ± 2 °C, then transferred into the growth chamber at 22 ± 2°C under a 12 h daylight photoperiod [43]. Petri dishes were controlled at one-day intervals for solutions content. Germinated seedlings were counted every two days for ten days. Seeds were considered to be germinated when radicles were at least 2 mm long (Figure 10).

At the end of the germination period, the germination percentage, rate of germination, and the mean germination time (MGT) under different salt concentration were calculated. The rate of germination was estimated by using a modified Timson index of germination velocity:

Germination rate = ∑ *GP*/*T* (1)

where GP is the percentage of seed germination at each day, and T is the total germination period [34]. Germination percentages were measured every day including the ending day using radicle expulsion (2 mm long) [56]. The final germination percentage (FGP), the cumulative percent of the total number of seeds germinated during the ten days over the total number of seeds tested for germination was calculated. The germination rate (GR), the number of days to reach 50% germination (average germination time, T50) was also determined, as it expresses the germination energy in terms of the depletion of the seed reserves.

### 4.3. Hydroponic Culture Assay under Salt Stress

The hydroponic assay for testing the effects of salinity on seedlings was conducted using the nutrient solution of [57]. For this test, 25 seeds (5 plants/*n* = 5 repetitions for each salt treatment) (nine populations) were germinated and monitored regularly until the appearance of the first trifoliate leaves, at which stage the salt stress was applied. The seedlings were first transplanted into test tubes containing the nutrient solution and different concentrations of NaCl (0, 50, 100, 150 and 200 mM) and then transferred into plastic containers containing the Hoagland’s saline solution and equipped with an aeration system. Salt stress was continued for three days.

### 4.4. Parameters Measured

Seedling shoot length (SL), seedling root length (RL), and dry weight of the whole plant (DW: mg plant^−1^), the parameters typically used for plant growth assessment under salt stress, were measured at the end of the stress period. The dry weights of each whole seedling’s weight were determined after drying samples at 70 °C in an oven until a constant weight was achieved.

### 4.5. Chlorophyll Content

Small lamina discs were cut from fresh apical leaves (100 mg of fresh samples), added to 10 mL of 95% acetone, and then kept in the dark at 4 °C for 48 h. Calculation of chlorophyll *a* (Chl *a*) and chlorophyll *b* (Chl *b*) contents was done by applying the following equations with the appropriate extinction coefficients [58]:

- Chl *a* (μg mL^−1^) = 12.25.·A663 − 2.79.·A647
- Chl *b* (μg mL^−1^) = 21.5.·A647 − 5.10.·A663
- Total Chlorophyll (μg mL^−1^) = (20.2 × A645) + (8.02 × A663)

A = absorbance at specific wavelength

### 4.6. Proline Content

Free proline was quantified following the protocol of [59]. Approximately 0.5 g of plant tissue was homogenized in 5 mL of 3 % aqueous sulphosalicylic acid filtered through filter paper. Two milliliters of the supernatant was heated with 2 mL of acid ninhydrin, and 2 mL of glacial acetic acid was added in a water bath at 100 °C for 1 h. The reaction was stopped in an ice bath. For extraction, 4 mL of toluene was added, and samples were mixed vigorously for 15 to 20 s. Samples were then set aside to allow separation of the organic and aqueous phases. The absorbance of the organic phase was measured at 520 nm with a UV-VIS spectrophotometer (UV-160, Shimadzu, Tokyo, Japan) using toluene as a blank. Proline concentration was determined from a standard curve, and the value was determined using calibration curved expressed based on fresh weight as µmol proline g^−1^ FW.

### 4.7. Total Sugars

The amount of reducing sugars was estimated by a modified method of [60]. One milliliter of alcoholic extract 80% neutral ethanol was mixed with 1.0 mL of a freshly prepared mixture of 25 parts of reagent “A” (4 g CuSO_4_, 5H_2_O, 24g anhydrous Na_2_CO_3_) and 1 part of reagent “B” (16 g Na-K-tartarate and 180 g anhydrous Na_2_SO_4_ dissolved in 1 L distilled water) (25:1). The samples were incubated in a boiling water bath for 20 min and cooled under running water. One milliliter of arsenomolybdate reagent (25 g Ammonium molybdate dissolved in 450 mL distilled water, 3 g sodium arsenate dissolved in 25 mL distilled water, 21 mL concentrated HCl) was added, and the color allowed to develop for 15 min. The mixture was then diluted to 10 mL and the absorbance at 500 nm was measured with a spectrophotometer. Reagent blanks were used to adjust absorbance to zero. Reducing sugar content was estimated by using a standard curve prepared with dextrose.

### 4.8. Salt Tolerance Index

This value of salt tolerance index (TI) was calculated as the ratio of the total dry weight of plants subjected to different salt concentrations to the total dry weight of control plants [41,61].

Salt tolerance index TI (%) = (TDW at Sx/TDW at S0) × 100(2)

where TDW = Total dry weight, S0 = control, and Sx = salt concentration.

Rather than comparing absolute growth rates, salinity tolerance is expressed as a relative reduction in the mass of material as a percentage of control, which indicates the relative vigor of the plant under salt stress conditions.

Sensitivity Index (SI%) reduction = 100 × ((salt treatment ‒ control)/control)(3)

SI(FGP): sensitivity index for germination

SI(DW): sensitivity index for seedling growth

### 4.9. Statistical Analysis

Physiological properties of plants in individual populations were compared using factorial ANOVA and Tukey’s post hoc test (*p* < 0.05) using the Statistica software ver. 12 (Statsoft Inc. 2012). Canoco 5 [62] was used for PCA (Principal Component Analysis) calculated from centered (not standardized) data. This analysis was appropriate for comparing differences between annual *Medicago* populations in response to salt stress.

## 5. Conclusions

To develop salt-tolerant varieties, it is necessary to establish an effective screening method that allows accurate identification of salt tolerance parameters useful for analysis at the molecular level for genetic breeding. Germination and the early growth stages would be the most sensitive phases that are affected by salinity. Varieties tolerating salinity at the germination usually continue resistance in later stages, but sprouting is not sufficient to identify salt stress-tolerant genotypes. Results showed a highly significant effect of salt stress, populations, and their interaction on the germination parameters. For biochemical parameters, the significant effect of salt on chlorophyll b, total chlorophyll, leaf soluble sugars content, and proline content, as well as a significant effect on chlorophyll a, were observed. The morphological parameters were greatly influenced by salinity exposure. Negative correlations were found between soluble root sugars and proline and between soluble leaf sugars and epicotyl length, while positive correlation was obtained between proline and plant weight. In addition, PCA analysis showed that the tolerant populations had longer epicotyls and radicles and produced more proline, while the sensitive ones were rich in sugars. According to these results, three populations of *M. ciliaris* (*Pop1*, *355*, and *667*) among the seven studied were found to be more salt tolerant and could be recommended as the most promising for germination and growth under moderate salt stress. Indeed, *Pop1* is not the most tolerant to salt stress during the germination stage, but it did have significant tolerance towards NaCl at the seedling stage. As different genetic mechanisms are likely involved in salt tolerance at the various developmental stages, *Pop1* may still serve as a valuable genetic resource for future breeding material. On the other hand, populations *306*, *773*, and *M. scut* were identified as moderately tolerant to sensitive to salt stress depending on salt rates.

Annual *Medicago* as pasture legumes can be sown in dry lands of arid and semi-arid regions of the world, where often soil salinity and climactic conditions limit the options available for forage production. Alfalfa from Northern Tunisia can be successfully raised in dry and rain-fed farming systems. Our results confirmed that the study of germination and early growth of spontaneous legumes may be used as an efficient strategy to reveal genetic variations in these plants that make them resistant to salt stress. This approach allows selection of desirable genotypes, thus enabling more efficient application of various breeding methods to achieve stress-tolerant annual alfalfa genotypes.

Further studies at later stages, such as the reproductive phase, and assessment of their performance under field conditions are needed to evaluate the effect of salt stress on yield. A deeper understanding of the genetic mechanisms through a transcriptomic approach would facilitate the selection of useful germplasm as sources for a salt stress tolerance breeding program. The *Pop1*, *355*, and *667* populations should be retained for their salt tolerance and should be tested at field conditions and under combined stresses such as salt and drought.

## Figures and Tables

**Table 1:** Final germination percentage (FGP) under salt stress (0, 50, 100, 150, 200 mM NaCl) of seven populations of *M. ciliaris* (*Pop1, Pop2, Pop3, 306, 355, 667, 773*), compared to one population of *M. intertexta* (*M. int*) and one population of *M. scutellata* (*M. scut*). The means are averages of 10 seeds and 5 replications. Different letters indicate in the same column a significant difference at *p* < 0.05, Tukey’s post hoc test.

Populations	Salt Concentration				
	0 mM	50 mM	100 mM	150 mM	200 mM
*Pop1*	98^a^	70^bc^	63^bc^	34^de^	22^bc^
*Pop2*	100^a^	100^a^	85^ab^	88^a^	24^bc^
*Pop3*	100^a^	66.7^bc^	57^c^	54^cd^	38.4^b^
*306*	95^ab^	85^ab^	65^c^	44^cde^	16^c^
*355*	80^bc^	86.7^ab^	83^abc^	78^ab^	21^bc^
*667*	62.5^d^	75^bc^	80^bc^	40^cde^	26.4^bc^
*773*	95^ab^	56.7^c^	63^bc^	42^cde^	30.4^bc^
*M. scut*	67.5^cd^	70^bc^	90^ab^	60^bc^	21^bc^
*M. int*	100^a^	100^a^	95^a^	46^e^	-

**Table 2:** Variance analysis for final germination percentage (FGP) of seven populations of *M. ciliaris* (*Pop1, Pop2, Pop3, 306, 355, 667, 773*), compared to one population of *M. intertexta* (*M. int*) and one population of *M. scutellata* (*M. scut*).

Factors	Average	Sum Square	Average Square	F	*p*
salt concentration(S)	60.7	179,795.5556	35,959.1111	180.05	<0.0001
Population (Pop)		15,020	1877.5000	9.40	<0.0001
Repetition (Rep)		739.2593	184.8148	0.93	0.4501
Salt×Pop		42,984.4444	1074.6111	5.38	<0.0001

**Table 3:** Germination rate (T50) under salt stress (0, 50, 100, 150, 200 mM NaCl) of seven populations of *M. ciliaris* (*Pop1, Pop2, Pop3, 306, 355, 667, 773*), one population of *M. intertexta* (*M. int*), and one population of *M. scutellata* (*M. scut*). The values are the averages of 10 seeds and five replications. Different letters indicate a significant difference at *p* < 0.05, Tukey’s post hoc test.

Population	NaCl Concentration				
	0 mM	50 mM	100 mM	150 mM	200 mM
*Pop1*	1.8^ab^	2.2^a^	1.8^ab^	1.2^a^	1.6^a^
*Pop2*	1.2^b^	1.6^a^	1^b^	1.4^a^	3^a^
*Pop3*	1.6^ab^	2.8^a^	3.6^a^	3.6^a^	2.6^a^
*306*	1.6^ab^	2.2^a^	3.6^a^	2.6^a^	1.6^a^
*355*	1.8^ab^	1.8^a^	1^d^	3.4^a^	1.2^a^
*667*	2^a^	2.2^a^	2.2^bc^	3^a^	2.6^a^
*773*	1.2^b^	1.2^a^	1.6^cd^	2.8^a^	1.4^a^
*M. scut*	1.8^ab^	1.6^a^	2.8^ab^	3.4^a^	3.4^a^
*M. int*	1.6^ab^	1.2^a^	1.2^cd^	2^a^	3.4^a^
Means of treatments (*n* = 10 seeds/5 repetitions)	1.7^b^	1.9^b^	2.3^ab^	2.6^a^	2.3^ab^

**Table 4:** The two-way ANOVA applied to growth parameters for seven populations of *M. ciliaris* (*Pop1, Pop2, Pop3, 306, 355, 667, 773*), compared to one population of *M. intertexta* (*M. int*) and one population of *M. scutellata* (*M. scut*). The means are averages of 25 plants.

Characteristic	MS Populations	MS Salt Stress	MS Interaction	F Population	F Salt Stress	F Interaction
DW g plant^−1^	0.004556	0.049997	0.002733	8.918****	97.872****	5.360****
Epicotyl (cm)	4.477	7.454	1.508	17.219****	28.670****	5.800****
Radicle (cm)	21.744	60.324	2.104	54.885****	152.262****	5.310****

MS = mean square, **** = highly significant, *p* < 0.0000000.

**Table 5:** Effect of salt stress (0, 50, 100, 150, 200 mM NaCl) on leaf chlorophyll content for seven populations of *M. ciliaris* (*Pop1, Pop2, Pop3, 306, 355, 667, 773*), compared to one population of *M. intertexta* (*M. int*) and one population of *M. scutellata* (*M. scut*). The means are averages of *n* = 5. The standard deviation annotated by different lower-case letters in the same stress type indicate significant differences at *p* < 0.05 according to Tukey’s post hoc test.

NaCl Concentration (mM)	Populations								
	*Pop1*	*Pop2*	*Pop3*	*306*	*355*	*667*	*773*	*M. int*	*M. Scut*
**Chl *a***									
0	1.09 ± 0.03^c^	0.72 ± 0.03^d^	0.52 ± 0.02^e^	2.34 ± 0.02^a^	0.3 ± 0.02^f^	1.21 ± 0.06^b^	0.57 ± 0.01^e^	0.34 ± 0.02^f^	0.53 ± 0.01^e^
50	0.71 ± 0.01^c^	0.48 ± 0.02^e^	0.24 ± 0.003^g^	1.17 ± 0.01^a^	0.60 ± 0.01^d^	0.58 ± 0.03^d^	1.14 ± 0.01^a^	0.78 ± 0.01^b^	0.29 ± 0.04^f^
100	1.23 ± 0.03^c^	0.53 ± 0.003^g^	0.24 ± 0.02^h^	0.68 ± 0.01^f^	0.93 ± 0.04^d^	0.76 ± 0.02^e^	1.30 ± 0.03^b^	1.57 ± 0.01^a^	0.49 ± 0.01^g^
150	0.72 ± 0.005^c^	0.73 ± 0.01^c^	0.36 ± 0.01^f^	0.61 ± 0.03^de^	0.57 ± 0.01^e^	1.37 ± 0.01^a^	0.92 ± 0.005^b^	0.24 ± 0.03^g^	0.65 ± 0.01^d^
200	0.36 ± 0.01^f^	1.11 ± 0.02^a^	0.15 ± 0.01^h^	0.26 ± 0.01^g^	0.49 ± 0.02^e^	0.87 ± 0.01^d^	0.96 ± 0.005^c^	1.03 ± 0.005^b^	0.48 ± 0.05^e^
**Chl *b***									
	0.47 ± 0.02^e^	0.12 ± 0.02^f^	0.69 ± 0.01^d^	0.70 ± 0.04^d^	0.74 ± 0.03^d^	1.49 ± 0.03^a^	1.20 ± 0.03^c^	1.370.05^b^	0.410.02^e^
50	0.19 ± 0.02^c^	0.19 ± 0.03^c^	0.42 ± 0.03^b^	0.95 ± 0.004^a^	0.45 ± 0.01^b^	0.13 ± 0.01^d^	0.92 ± 0.02^a^	0.13 ± 0.01^d^	0.15 ± 0.01^dc^
100	1.15 ± 0.05^a^	0.77 ± 0.02^b^	0.34 ± 0.01^d^	0.52 ± 0.04^c^	0.25 ± 0.03^e^	0.42 ± 0.07^d^	0.76 ± 0.03^b^	0.34 ± 0.01^d^	0.15 ± 0.04^f^
150	0.17 ± 0.01^d^	0.52 ± 0.02^c^	0.03 ± 0.01^d^	0.75 ± 0.09^b^	2.24 ± 0.18^a^	0.77 ± 0.01^b^	0.080.01^d^	0.21 ± 0.03^d^	0.130.02^d^
200	0.149 ± 0.01^a^	1.16 ± 0.02^bc^	0.50 ± 0.02^e^	0.59 ± 0.03^e^	1.16 ± 0.08^bc^	0.50 ± 0.02^e^	1.25 ± 0.01^b^	1.11 ± 0.01^c^	0.74 ± 0.01^d^
***Tot* Chl**									
0	1.56 ± 0.02^e^	0.83 ± 0.01^i^	1.21 ± 0.04^f^	3.04 ± 0.02^a^	1.04 ± 0.01^g^	2.71 ± 0.02^b^	1.77 ± 0.03^c^	1.71 ± 0.02^d^	0.94 ± 0.03^h^
50	0.90 ± 0.01^d^	0.77 ± 0.003^f^	0.66 ± 0.03^f^	2.12 ± 0.01^a^	1.05 ± 0.003^c^	0.71 ± 0.02^e^	2.05 ± 0.01^b^	0.91 ± 0.01^d^	0.44 ± 0.01^g^
100	2.38 ± 0.02^a^	1.31 ± 0.02^d^	0.76 ± 0.01^f^	1.20 ± 0.03^c^	1.18 ± 0.01^e^	1.18 ± 0.04^e^	2.06 ± 0.04^b^	1.99 ± 0.04^c^	0.64 ± 0.04^g^
150	0.88 ± 0.01^de^	1.25 ± 0.005^c^	0.39 ± 0.005^f^	1.36 ± 0.09^c^	2.86 ± 0.17^a^	2.14 ± 0.01^b^	1 ± 0.005^d^	0.45 ± 0.01^f^	0.79 ± 0.01^c^
200	1.86 ± 0.01^f^	2.27 ± 0.05^b^	0.65 ± 0.02^ef^	0.85 ± 0.06^d^	1.65 ± 0.06^de^	1.37 ± 0.02^a^	2.21 ± 0.01^c^	2.14 ± 0.01^b^	1.22 ± 0.01^c^

**Table 6:** Geographical origins and characteristics of the studied populations of *Medicago ciliaris*, *Medicago scutellata*, and *Medicago intertexta* [55].

Species/Populations	Code /NGBT*	Province	Alt (m)	AAR*** (mm)	Tmax (°C)	Tmin (°C)	CE (mM/cm)
*Medicago ciliaris*							
*Pop1*	9151	Raoued El Hessiane	5.75	398.5	35.1	5.4	0.39
*Pop2*	9152	Kalaat El Andalous	5.85	398.5	35.1	5.4	0.41
*Pop3*	9153	Ghar El Melh	6.19	497.8	32.9	6.7	0.16
*306*	9141	Siliana	575	341.1	38.6	1.6	0.27
*355*	9142	Dougga	320	493.5	21.7	9.9	0.24
*667*	9143	Zaghouan	300	496	32.6	6.7	2.41
*773*	9144	Mateur 1	5	527.1	33.4	4.9	0.74
*Medicago scutellata*	Wafra*	Saîda/Manouba	114	450	45	6	-
Medicago intertexta**	-	SidiAissa/Nabeul	120	358	36	8.4	-

* The National Gene Bank (NGB) of Tunisia | *** AAR: Average Annual Rainfall | ** variety registered in the catalogue of plant varieties gene bank.

## References

[1] Shokat S., Großkinsky D.K. (2019). Tackling salinity in sustainable agriculture-What developing countries may learn from approaches of the developed world. Sustainability.

[2] Minhas P.S., Ramos T.B., Ben-Gal A., Pereira L.S. (2020). Coping with salinity in irrigated agriculture: Crop evapotranspiration and water management issues. Agric. Water Manag..

[3] Jacobsen S.E., Jensen C.R., Liu F. (2012). Improving crop production in the arid Mediterranean climate. Field Crop. Res..

[4] Srivastava N. (2020). Reclamation of Saline and Sodic Soil Through Phytoremediation. Environmental Concerns and Sustainable Development.

[5] Chaves M.M., Flexas J., Pinheiro C. (2009). Photosynthesis under drought and salt stress: Regulation mechanisms from whole plant to cell. Ann. Bot..

[6] Louati D., Majdoub R., Rigane H., Abida H. (2018). Effects of Irrigating with Saline Water on Soil Salinization (Eastern Tunisia). Arab. J. Sci. Eng..

[7] Mbarki S., Sytar O., Cerda A., Zivcak M., Rastogi A., He X., Zoghlami A., Abdelly C., Brestic M. (2018). Strategies to mitigate the salt stress effects on photosynthetic apparatus and productivity of crop plants. Salinity Responses and Tolerance in Plants, Volume 1: Targeting Sensory, Transport and Signaling Mechanisms.

[8] Safdar H., Amin A., Shafiq Y., Ali A., Yasin R. (2019). A review: Impact of salinity on plant growth. Nat. Sci..

[9] Acosta-motos J.R., Penella C., Hernández J.A., Sánchez-blanco M.J., Navarro J.M., Gómez-bellot M.J. (2020). Towards a Sustainable Agriculture: Strategies Involving Phytoprotectants against Salt Stress. Agronomy.

[10] Kumar A., Kumar A., Mann A., Devi G., Sharma H., Singh R., Sanwal S.K. (2019). Phytoamelioration of the Salt-Affected Soils Through Halophytes. Ecophysiology, Abiotic Stress Responses and Utilization of Halophytes.

[11] Ismail S., Rao N.K., Dagar J.C. (2019). Identification, Evaluation, and Domestication of Alternative Crops for Saline Environments. Research Developments in Saline Agriculture.

[12] Abiala M.A., Abdelrahman M., Burritt D.J., Tran L.S.P. (2018). Salt stress tolerance mechanisms and potential applications of legumes for sustainable reclamation of salt-degraded soils. Land Degrad. Dev..

[13] Mbarki S., Cerdà A., Zivcak M., Brestic M., Rabhi M., Mezni M., Jedidi N., Abdelly C., Pascual J.A. (2018). Alfalfa crops amended with MSW compost can compensate the effect of salty water irrigation depending on the soil texture. Process. Saf. Environ. Prot..

[14] Morton M.J.L., Awlia M., Al-Tamimi N., Saade S., Pailles Y., Negrão S., Tester M. (2019). Salt stress under the scalpel-dissecting the genetics of salt tolerance. Plant. J..

[15] Venâncio C., Pereira R., Lopes I. (2020). The influence of salinization on seed germination and plant growth under mono and polyculture. Environ. Pollut..

[16] Hernández J.A. (2019). Salinity tolerance in plants: Trends and perspectives. Int. J. Mol. Sci..

[17] Kotula L., Kwa H.Y., Nichols P.G.H., Colmer T.D. (2019). Tolerance and recovery of the annual pasture legumes Melilotus siculus, Trifolium michelianum and Medicago polymorpha to soil salinity, soil waterlogging and the combination of these stresses. Plant. Soil.

[18] Smith A.P., Moore A.D. (2020). Whole farm implications of lucerne transitions in temperate crop-livestock systems. Agric. Syst..

[19] Nedjimi B., Zemmiri H. (2019). Salinity Effects on Germination of Artemisia herba–alba Asso: Important Pastoral Shrub from North African Rangelands. Rangel. Ecol. Manag..

[20] Natasha K. (2019). Effect of sodium chloride, potassium chloride on germination and growth of Foxtail millet (*Setaria italica* L.). Pure Appl. Biol..

[21] Bouslama M., Denden M., Hajlaoui H. (2007). Etude de la variabilité intraspécifique de tolérance au stress salin du pois chiche (*Cicer arietinum* L.) au stade germination. Tropicultura.

[22] Ouerghi K., Abdi N., Maazaoui H., Hmissi I., Bouraoui M., Sifi B. (2016). Physiological and morphological characteristics of pea (Pisum sativum L.) seeds under salt stress. J. New Sci..

[23] Debez A., Ben Slimen I.D., Bousselmi S., Atia A., Farhat N., El Kahoui S., Abdelly C. (2019). Comparative analysis of salt impact on sea barley from semi-arid habitats in Tunisia and cultivated barley with special emphasis on reserve mobilization and stress recovery aptitude. Plant. Biosyst..

[24] Rahman M.M., Mostofa M.G., Rahman M.A., Miah M.G., Saha S.R., Karim M.A., Keya S.S., Akter M., Islam M., Tran L.S.P. (2019). Insight into salt tolerance mechanisms of the halophyte Achras sapota: An important fruit tree for agriculture in coastal areas. Protoplasma.

[25] Zulfiqar F., Akram N.A., Ashraf M. (2020). Osmoprotection in plants under abiotic stresses: New insights into a classical phenomenon. Planta.

[26] Nawaz K., Hussain K., Majeed A., Khan F., Afghan S., Ali K. (2010). Fatality of salt stress to plants: Morphological, physiological and biochemical aspects. Afr. J. Biotechnol..

[27] Bayuelo-Jiménez J.S., Craig R., Lynch J.P. (2002). Salinity tolerance of Phaseolus species during germination and early seedling growth. Crop. Sci..

[28] Rashid M.H.O., Islam S., Bari M. (2019). In vitro screening for salt stress tolerance of native and exotic potato genotypes by morphological and physiological parameters. J. Bio-Sci..

[29] Taffouo V., Meguekam L., Kenne M., Yayi E., Magnitsop A., Akoa A., Ourry A. (2009). Germination et accumulation des métabolites chez les plantules de légumineuses cultivées sous stress salin. Agron. Afr..

[30] Ibrahim M.H., Abas N.A., Zahra S.M. (2019). Impact of Salinity Stress on Germination of Water Spinach (Ipomoea aquatica). Annu. Res. Rev. Biol..

[31] Lovato M.B., Martins P.S. (1997). Genetic variability in salt tolerance during germination of Stylosanthes humilis H.B.K. and association between salt tolerance and isozymes. Braz. J. Genet..

[32] Kayess M.O., Rahman M.L., Ahmed K., Khan M.R., Hossan M.S., Hossain M.S., Khanam M.M., Chandra Pal D. (2020). Effect of Salinity Stress on Different Root and Shoot Traits of Selected Tomato Cultivars. Asian J. Adv. Res. Rep..

[33] Farissi M., Ghoulam C., Bouizgaren A. (2013). Changes in water deficit saturation and photosynthetic pigments of Alfalfa populations under salinity and assessment of proline role in salt tolerance. Agric. Sci. Res. J..

[34] Moghaddam M., Farhadi N., Panjtandoust M., Ghanati F. (2019). Seed germination, antioxidant enzymes activity and proline content in medicinal plant Tagetes minuta under salinity stress. Plant. Biosyst..

[35] Pujol J.A., Calvo J.F., Ramírez-Díaz L. (2000). Recovery of germination from different osmotic conditions by four halophytes from southeastern Spain. Ann. Bot..

[36] Mirza H., Kamrun N., Masayuki F., Hirosuke O., Islam T.M., Shahzad B., Fahad S., Tanveer M., Saud S., Khan I.A. (2019). Plant Responses and Tolerance to Salt Stress. Approaches for Enhancing Abiotic Stress Tolerance in Plants.

[37] Shoukat E., Ahmed M.Z., Abideen Z., Azeem M., Ibrahim M., Gul B., Khan M.A. (2020). Short and long term salinity induced differences in growth and tissue specific ion regulation of Phragmites karka. Flora Morphol. Distrib. Funct. Ecol. Plants.

[38] Chartzoulakis K., Klapaki G. (2000). Response of two greenhouse pepper hybrids to NaCl salinity during different growth stages. Sci. Hortic. Amst..

[39] Mahdavi B., Sanavy S.A.M.M. (2007). Germination and seedling growth in grasspea (Lathyrus sativus) cultivars under salinity conditions. Pak. J. Biol. Sci..

[40] Li R., Shi F., Fukuda K., Yang Y. (2010). Effects of salt and alkali stresses on germination, growth, photosynthesis and ion accumulation in alfalfa (*Medicago*
*sativa* L.). Soil Sci. Plant. Nutr..

[41] Ranjbar G.H. (2012). Anagholi, a Evaluation of physiological indices of salinity tolerance in forage Sorghum (Sorghum bicolor) lines. Int. Res. J. Appl. Basic Sci..

[42] Benabderrahim M.A., Guiza M., Haddad M. (2020). Genetic diversity of salt tolerance in tetraploid alfalfa (*Medicago*
*sativa* L.). Acta Physiol. Plant..

[43] Bhatt A., Bhat N.R., Santo A., Phartyal S.S. (2019). Influence of Temperature, Light and Salt on the Germination of Deverra Triradiata Seeds. Seed Sci. Technol..

[44] Bukhari S.A.H., Peerzada A.M., Javed M.H., Dawood M., Hussain N., Ahmad S. (2019). Growth and Development Dynamics in Agronomic Crops Under Environmental Stress. Agronomic Crops.

[45] Thouraya R., Imen I., Imen H., Riadh I., Ahlem B., Hager J. (2013). Effet du stress salin sur le comportement physiologique et métabolique de trois variétés de piment (*Capsicum annuum* L.). J. Appl. Biosci..

[46] Wu H., Guo J., Wang C., Li K., Zhang X., Yang Z., Li M., Wang B. (2019). An effective screening method and a reliable screening trait for salt tolerance of Brassica napus at the germination stage. Front. Plant. Sci..

[47] Naeem M.S., Jin Z.L., Wan G.L., Liu D., Liu H.B., Yoneyama K., Zhou W.J. (2010). 5-Aminolevulinic acid improves photosynthetic gas exchange capacity and ion uptake under salinity stress in oilseed rape (Brassica napus L.). Plant. Soil.

[48] Poór P., Czékus Z., Ördög A. (2019). Role and Regulation of Glucose as a Signal Molecule to Salt Stress. Plant Signaling Molecules.

[49] Jawahar G., Rajasheker G., Maheshwari P., Punita D.L., Jalaja N., Kumari P.H., Kumar S.A., Afreen R., Karumanchi A.R., Rathnagiri P. (2019). Osmolyte Diversity, Distribution, and Their Biosynthetic Pathways. Plant Signaling Molecules.

[50] Lucho S.R., do Amaral M.N., Auler P.A., Bianchi V.J., Ferrer M.Á., Calderón A.A., Braga E.J.B. (2019). Salt Stress-Induced Changes in In Vitro Cultured Stevia rebaudiana Bertoni: Effect on Metabolite Contents, Antioxidant Capacity and Expression of Steviol Glycosides-Related Biosynthetic Genes. J. Plant. Growth Regul..

[51] Hamrouni L., Hanana M., Abdelly C., Ghorbel A. (2011). Exclusion du chlorure et inclusion du sodium: Deux mécanismes concomitants de tolérance à la salinité chez la vigne sauvage Vitis vinifera subsp. sylvestris (var. ’séjnène’). Biotechnol. Agron. Soc. Environ..

[52] Shafi A., Zahoor I., Mushtaq U. (2019). Proline Accumulation and Oxidative Stress: Diverse Roles and Mechanism of Tolerance and Adaptation Under Salinity Stress. Salt Stress, Microbes, and Plant Interactions: Mechanisms and Molecular Approaches.

[53] Hasanuzzaman M., Fujita M., Oku H., Islam M.T., Khanna-Chopra R., Kumar Semwal V., Lakra N., Pareek A. (2019). Proline—A Key Regulator Conferring Plant Tolerance to Salinity and Drought. Plant Tolerance to Environmental Stress.

[54] Forlani G., Bertazzini M., Cagnano G. (2019). Stress-driven increase in proline levels, and not proline levels themselves, correlates with the ability to withstand excess salt in a group of 17 Italian rice genotypes. Plant. Biol..

[55] Cheima J. (2012). Genetic diversity of pod traits in local populations of *Medicago ciliaris* L.. Iosr J. Agric. Vet. Sci..

[56] Roberts S.J., Koenraadt H. (2020). Validated Seed Health Testing Methods. International Rules for Seed Testing.

[57] Hoagland D.R., Arnon D.I. (1950). The Water-Culture Method for Growing Plants Without Soil.

[58] Lichtenthaler H.K. (1987). Chlorophylls and Carotenoids: Pigments of Photosynthetic Biomembranes. Methods Enzymol..

[59] Bates L.S., Waldren R.P., Teare I.D. (1973). Rapid determination of free proline for water-stress studies. Plant. Soil.

[60] Do B.C., Dang T.T., Berrin J.G., Haltrich D., To K.A., Sigoillot J.C., Yamabhai M. (2009). Cloning, expression in Pichia pastoris, and characterization of a thermostable GH5 mannan endo-1,4-beta-mannosidase from Aspergillus niger BK01. Microb. Cell Fact..

[61] El Goumi Y., Fakiri M., Lamsaouri O., Benchekroun M. (2014). Salt stress effect on seed germination and some physiological traits in three Moroccan barley (Hordeum vulgare L.) cultivars. J. Mater. Environ. Sci..

[62] Smilauer P., Leps J. (2014). Multivariate Analysis of Ecological Data Using Canoco 5.

